# Male Breast Cancer Presenting as Nipple Discharge

**DOI:** 10.1155/2011/804843

**Published:** 2011-12-05

**Authors:** A. Farooq, K. Horgan

**Affiliations:** Department of Surgery, Leeds General Infirmary, Leeds Teaching Hospitals NHS Trust, Leeds LS1 3BR, UK

## Abstract

Male breast cancer (MBC) is a rare disease and constitutes less than 1% of all breast carcinoma cases. Although MBC most often presents with a palpable mass, failure to recognise the significance of other symptoms may lead to a delay in diagnosis. Nipple discharge (ND) is a rare symptom in men, but it may herald an underlying malignancy. We present two cases of (MBC) presenting with ND and emphasise the importance of this clinical sign in suspecting underlying malignancy and an opportunity for early diagnosis. We also discuss the clinical significance of ND in men in relation to current literature.

## 1. Introduction

Male breast cancer (MBC) is a rare disease accounting for less than 1% of all breast cancers [1, 2]. In central African countries, there is a much higher proportion of cases of MBC but the reasons for this geographical variation are unclear [3, 4]. The incidence of both male and female breast cancer seems to be rising [5]. While this trend may simply reflect increasing life expectancy, it is likely that other explanations exist. The average age at diagnosis of MBC is 68 years, which is older than the typical presentation of female breast cancer at mean age of 58 years. Furthermore, MBC has a unimodal age distribution as opposed to female breast cancer which has a bimodal peak at age 52 and 71 [2, 6–8]. This is not necessarily due to a biological cause but is likely to be related to the onset of breast screening in women at age 50.

Literature on MBC is limited due to its rarity, and management is largely based on evidence from data of female breast cancer. However, MBC is not exactly the same as female breast cancer [7, 8]. Although most cases present as a palpable mass, it is imperative to be aware of other signs and symptoms of MBC so that the disease may potentially be detected at an earlier stage conferring a survival benefit to the patient [9]. One such sign of underlying MBC is nipple discharge (ND). Data on ND in men in the current literature, however, is very limited. We present two cases of MBC presenting with ND and discuss the clinical significance of this sign in men.

## 2. Case Report

(1) A healthy 87-year-old man presented with a three-month history of spontaneous, clear, left-sided ND. There was a significant family history of breast cancer as a maternal aunt, and two sisters were diagnosed in their early 60's. On examination, he had a firm, 2 cm mass in the upper outer quadrant of the left breast. There was no skin ulceration or tethering, no nipple retraction, and there were no enlarged axillary lymph nodes. The discharge came from the centre of the nipple, and, clinically, it was not possible to say if this was uniductal. The discharge was sent for cytology, and a core biopsy of the mass was also performed. ND cytology was inconclusive with no evidence of any epithelial cells, but core biopsy of the lump revealed the presence of invasive adenocarcinoma.

After counselling, the patient went on to have a mastectomy and axillary sentinel lymph node biopsy (SLNB). Histology showed a grade 2 invasive papillary carcinoma 19 mm in maximum dimension, with clear margins. No lymphovascular invasion was seen, and the one SLNB was negative for metastatic tumour. Immunohistochemistry demonstrated the tumour was strongly oestrogen receptor (ER) positive without HER2 over amplification. The patient made a good postoperative recovery and had adjuvant endocrine treatment with tamoxifen. Due to his strong family history, he was offered genetic counselling but he refused and remained well 2 years postoperatively.

(2) A 77-year-old gentleman presented with bloody ND and a lump in the left breast for 6 weeks. There was no history of trauma and no family history of note. On examination, there was a cord-like mass beneath the areola extending into the upper outer quadrant of the left breast, with no skin or nipple abnormality. The patient underwent a mammogram and ultrasound scan both of which were highly suggestive that the mass was malignant. The ND cytology revealed atypical epithelial cells. Core biopsy of the mass revealed the presence of invasive adenocarcinoma. The patient was treated with mastectomy and SLNB. Histology revealed a grade 2 invasive ductal carcinoma with clear margins and no lymphovascular invasion present. A single node was retrieved at SLNB which was negative for metastatic carcinoma. Immunohistochemistry showed the tumour was ER positive. He was prescribed adjuvant tamoxifen and remained well 4 years postoperatively.

## 3. Discussion

MBC is often diagnosed at a later stage than in women with more than 40% of men presenting with stage III or IV disease [10]. Published series have also consistently reported long median time intervals between onset of symptoms and diagnosis of MBC [10, 11]. Prolonged duration of symptoms and advanced stage at presentation is important as it correlates with decreased survival [11]. Delays in diagnosis of MBC are likely to result from a lack of awareness of the risk of MBC and the signs and symptoms that may indicate an underlying malignancy. Although the typical presentation of MBC in 75% of patients is a painless, firm, retroareolar breast lump, it is important to recognise the other less obvious signs and symptoms of MBC including nipple retraction, ulceration, Paget's disease of the nipple, axillary lymphadenopathy, breast pain, and ND [10–12]. 

ND alone is an uncommon presenting complaint in men, and published data is limited. Morrogh and King from the Memorial Sloane Kettering Centre, New York, presents the only significant series of MBC patients presenting with ND [13]. They found that among 430 patients who presented to their institution with ND over a 10-year period only 3% were male. However, 57% of these men presenting with ND had an underlying malignancy. This is in contrast to the female population in whom only 16% of patients who presented with ND had an underlying malignancy. Other smaller studies looking at the presence of ND in association with a palpable mass have found cancer rates of between 15–75% [14, 15]. Therefore, although ND of the male breast is uncommon, it warrants detailed evaluation due to its strong association with underlying malignancy.

Morrogh and King also found a significant delay in presentation in men who present with ND compared to those who present with a palpable mass. The median time interval between onset of symptoms and diagnosis was 16 weeks for patients presenting with ND and 3 weeks for those presenting with a palpable mass, raising the possibility that had this group recognised the significance of the ND they may have presented at an earlier stage [13]. 

For a male presenting with ND, the diagnostic pathway follows the same principles for breast cancer in women which is based on triple assessment. Clinical suspicion of malignancy is confirmed by clinical examination. Approximately half of men with a primary presenting complaint of ND have an underlying palpable mass [13]. Additional investigations such as mammography and targeted ultrasound may assist in diagnosis. However, definitive diagnosis relies on pathological assessment either with nipple fluid cytology, fine needle aspiration cytology, or core biopsy of a mass. 

The use of nipple fluid cytology to distinguish between patients with cancer and those with a benign ND has been a subject of ongoing debate. Current data suggest that ND cytological examination is only useful when positive and can have a false-negative rate for cancer of up to 50% [16, 17]. In our two patients, both underwent ND cytology, with cytology detecting suspicious malignant epithelial cells in one of the two patients. Although there are case reports describing the diagnosis of MBC on the basis of nipple cytology alone [17], the overall clinical utility of this investigation is questionable.

Although a high proportion of male patients presenting with ND will have an underlying malignancy, approximately 43% have a benign cause for their ND [13]. A number of benign causes for male ND have been described in the literature [18–21]. Duct ectasia is benign dilatation and shortening of the terminal ducts within 3 cm of the nipple. It is a common cause of ND in women increasing in incidence with age but rare in the male breast. Tedeschi and McCarthy reported the first male case in 1974 since when only a handful of cases have been reported in the literature [22]. Recently, duct ectasia has been reported in a man in association with the human immunodeficiency virus infection and Bechet's disease suggesting a possible immune mechanism being responsible [21]. Papillomas are characterised by formation of epithelial fronds that have both a luminal epithelial and outer myoepithelial cell layers supported by a fibrovascular core. The epithelial component can be subject to a spectrum of morphological changes ranging from metaplasia to hyperplasia atypical intraductal hyperplasia, and in situ carcinoma. Papillomas are again common in the female population and the commonest cause for bloody ND, but only a handful of cases occurring in men have been reported in the literature [19]. Most recently, two cases of intracystic papilloma causing ND in males on long-term phenothiazine treatment for schizophrenia and elevated prolactin levels have been reported [20]. Other more rare causes for benign ND in men include fibrocystic change, gynaecomastia, and various kinds of skin adnexal tumours arising in breast tissue [18–23].

In conclusion, ND in men is rare but when present carries a high probability of underlying malignancy. In the absence of screening mammography, we rely on the presence of clinical symptoms to detect MBC. Prognosis is largely determined by the stage at diagnosis; thus, any delay in presentation or diagnosis may reduce survival. By recognising subtle clinical features of early disease such as ND, there may be a window of opportunity to improve outcomes for male patients. An increased awareness of the significance of ND as an important symptom in men must therefore be highlighted to physicians and patients alike.
